# Observation of Communication by Physical Education Teachers: Detecting Patterns in Verbal Behavior

**DOI:** 10.3389/fpsyg.2018.00334

**Published:** 2018-03-19

**Authors:** Abraham García-Fariña, F. Jiménez-Jiménez, M. Teresa Anguera

**Affiliations:** ^1^Department of Specific Didactics, Faculty of Education, University of La Laguna, Santa Cruz de Tenerife, Spain; ^2^Faculty of Psychology, Institute of Neurosciences, University of Barcelona, Barcelona, Spain

**Keywords:** communicative strategies, social constructivism, systematic observation, physical education, instructional communication

## Abstract

The aim of this study was to analyze the verbal behavior of primary school physical education teachers in a natural classroom setting in order to investigate patterns in social constructivist communication strategies before and after participation in a training program designed to familiarize teachers with these strategies. The participants were three experienced physical education teachers interacting separately with 65 students over a series of classes. Written informed consent was obtained from all the students' parents or legal guardians. An indirect observation tool (ADDEF) was designed specifically for the study within the theoretical framework, and consisted of a combined field format, with three dimensions, and category systems. Each dimension formed the basis for building a subsequent system of exhaustive and mutually exclusive categories. Twenty-nine sessions, grouped into two separate modules, were coded using the Atlas.ti 7 program, and a total of 1991 units (messages containing constructivist discursive strategies) were recorded. Analysis of intraobserver reliability showed almost perfect agreement. Lag sequential analysis, which is a powerful statistical technique based on the calculation of conditional and unconditional probabilities in prospective and retrospective lags, was performed in GSEQ5 software to search for verbal behavior patterns before and after the training program. At both time points, we detected a pattern formed by requests for information combined with the incorporation of students' contributions into the teachers' discourse and re-elaborations of answers. In the post-training phase, we detected new and stronger patterns in certain sessions, indicating that programs combining theoretical and practical knowledge can effectively increase teachers' repertoire of discursive strategies and ultimately promote active engagement in learning. This has important implications for the evaluation and development of teacher effectiveness in practice and formal education programs.

## Introduction

Analysis of patterns in instructional communication allows teachers to reflect on their use of discursive strategies, check that these are aligned with their teaching goals, and resolve to incorporate them as a strategic part of their teaching.

Instructional communication patterns have been detected in the teaching of science (Cazden, 1988; Lemke, 1990) and mathematics (Lobato et al., 2005) and include the initiation-response-evaluation (IRE) pattern and the elicitation-response-evaluation (ERE) pattern (Bowers and Nickerson, 2001). Both patterns, or sequences, begin with a question designed to actively engage the students in the construction of knowledge. Nonetheless, it has been claimed that IRE sequences can deny students and teachers the opportunity for debate and negotiation (Wright and Forrest, 2007).

Social constructivism theory (Vygotsky, 1978) attaches great importance to dialogue between the agents engaged in the teaching and learning process. The general principle underlying this theory is that students can be helped to build knowledge by stimulating their higher mental processes through language-mediated interaction with their social and cultural environments. Edwards and Mercer (1989), claim that the value of educational discourse lies above all in its potential as a tool for negotiating students' previous representations and using these as scaffolding to build new knowledge throughout teacher-student interactions. This idea that language, as a modulator of an interactive system, influences cognitive and perceptual processes has also been highlighted by Lupyan (2012).

According to Coll and Onrubia (2001), instructional communication, which they refer to as “discursive strategies,” can serve three important pedagogical functions. It can (a) lead to the establishment of an initial platform for shared representations, where students' previous knowledge can be linked to the learning objective through discursive strategies involving questions or references to specific or social frameworks; (b) help students to adopt a positive attitude to learning through the use of meta-statements, incorporation of student contributions into their discourse, and characterization of knowledge as something shared; and (c) increase students' knowledge by guiding them toward increasingly complex representations. To achieve this, teachers can adopt a range of discursive strategies, such as re-elaboration of student contributions, categorization and labeling of certain aspects of content or context, abbreviation of expressions, modification of references used to talk about content, and use of recapitulation, summaries, and synthesis. By incorporating these and similar discursive strategies, which are defined by Coll and Onrubia (2001, p. 24) as a particular form of verbal communication used to guide the construction of knowledge, teachers can increase the impact and effectiveness of their instructional communication. Constructivist strategies are a valuable methodological resource, and they acquire meaning in context and at a given moment during a class. In a study on how to develop tools for an effective classroom, Powell and Kalina (2009) claimed that teachers need to use constructivist strategies and resources, such as examples linked to the topic being taught, questions to assess learning, and discussion and dialogue to recapitulate.

Several authors have analyzed social constructivism in the field of physical education through a theoretical lens. Constructivist physical educators value students' contributions, actively involve them in the construction of knowledge, and draw parallels between what is being taught and the students' personal experiences (Azzarito and Ennis, 2003). The main principles underlying the social constructivism theory (higher mental processes, language, mediation, cultural influence, and zone of proximal development) can all be applied to physical education, which involves teaching and learning about the development of motor skills and higher mental processes while enabling the exploration of concepts through action and language (Ussher and Gibbes, 2002). Authors such as Rovegno and Dolly (2006) and Ussher and Gibbes (2002) have also analyzed the constructivist perspective underlying diverse physical and sport education models, including the Teaching Games for Understanding (TGfU) and the sport education, personal and social responsibility, and adventure-based learning models. In all these models, dialogue between teachers and students regarding actions is critical.

The emergence of new sport education models centered around the intentional use of communicative strategies has had an important role in the creation of constructivist understanding (Morgan and Kingston, 2008). The TGfU model, considered by Light (2008) to be a good example of a social constructivist approach to teaching physical education, is perhaps the best-known example (Bunker and Thorpe, 1982; Kirk and MacPhail, 2002; Oslin and Mitchell, 2006). This model stresses the importance of using questions as a key communicative strategy for promoting reflection and tactical awareness among players, and accordingly, stimulates teachers' interest in the verbal behavior of students in relation to the meaning they attribute to the actions they perform (Wallian and Chang, 2007). As the TGfU model is built on problem-solving activities, high-quality questions are critical. These need to be planned and carefully constructed to ensure that they prompt critical thinking and favor the development of problem-solving skills (Dyson et al., 2004; Mitchell et al., 2006; Hubball et al., 2007). Questions addressed to the group help the students as a whole to scaffold knowledge, creating a learning environment that engages the students in the construction of knowledge (Harvey and Light, 2015) and helps them to learn to learn (Light, 2014). In teaching models that use a similar approach to the TGfU model, eliciting information from students in the form of questions is considered a key discursive strategy for building knowledge. Rink (1998) considers that “instructional strategies” used in the teaching of physical education (e.g., questions, references to existing knowledge, linking to other topics, and recapitulations) are themselves a methodological resource.

Webster (2010) proposed six skills that physical educators should master in order to improve the effectiveness of their instructional communication processes and increase student motivation. The first three are rhetorical communication skills (being clear, content relevance, and using humor) (Chesebro and Wanzer, 2006), while the second three are relational communication skills (immediacy, communication style and listening). For each of these skills, Webster proposed a series of specific instructional strategies.

Other studies in the field of physical education have analyzed the communication of content relevance. Webster et al. (2012), for example, analyzed the different ways in which teachers communicated content relevance and also the frequency with which they reported doing so according to whether they were expert or novices. Webster et al. (2011, 2013), in turn, analyzed how students perceived this communication of content relevance. The results showed that expert teachers communicate content relevance more frequently and that this strategy appears to instill in students a desire to keep learning. Other studies have analyzed instructional communication among physical educators from the perspective of need-supportive interactions (Haerens et al., 2013). Finally, a study of middle-school students' perceptions of instructional choices by physical education teachers found that these choices appeared to satisfy autonomy needs and promote student engagement (Agbuga et al., 2016). Overall, the different studies undertaken in this area show that the communication strategies (Anguera and Izquierdo, 2006) employed by physical educators have a significant effect on different aspects of learning.

The main aim of this study was to investigate whether it was possible to detect patterns in instructional communication strategies used by primary school physical education teachers. A secondary aim was to determine whether participation in a training intervention designed to teach social constructivist communication skills led to changes in practice.

## Materials and methods

### Design

To investigate the presence of constructivist discursive strategies (Coll and Onrubia, 2001), we designed a systematic observation study (Anguera, 2003; Castañer et al., 2016, 2017; Anguera et al., 2017) based on indirect observation (Lacy and Darst, 1985; Allison, 1990; Eckrich et al., 1994; Coleman and Mitchell, 2001; Anguera et al., 2018) to analyze the verbal behavior of physical education teachers in a natural classroom setting.

The nature and requirements of the study justified the use of a Nomothetic/Follow-up/Multidimensional design, which corresponds to quadrant IV of the observational methodology designs (Blanco-Villaseñor et al., 2003; Sánchez-Algarra and Anguera, 2013). The design was: (a) nomothetic because we analyzed the instructional communication, or verbal behavior, of three physical education teachers acting individually; (b) “follow-up” because we collected data over a series of successive sessions (intersessional follow-up) and also recorded each session in full, without interruption (intrasessional follow-up); and c) “multidimensional,” because although we were investigating just one overall response level or dimension (i.e., the teachers' instructional communication), the ad hoc observation instrument, which was derived from Coll and Onrubia (2001) social constructivist framework, unveiled three levels of response or dimensions (see description of observation instrument).

To investigate changes in the patterns detected following participation in a training activity focused on discursive strategies from a constructivist approach, we organized a collaborative action research program designed to familiarize physical education teachers with the use and value of these strategies as a methodological resource. Collaborative action research programs are accredited models (Carr and Kemmis, 1986; Elliott, 1991) that encourage interpretation and critical thinking to help teachers to reflect on and evaluate their practices and introduce changes that will make these more effective. The collaborative action research program designed for the present study was held over a 4-month period and was led by the first author. The program consisted of eight sessions, held every 2 weeks. It was held in the period between the teaching of the first and second modules. The participants learnt about and discussed social constructivist strategies and alternatives, and reflected on how these could improve their teaching. Because the collaborative action research program was interpreted as a training event, we use the terms “pre-training” and “post-training” in our presentation of data and results.

### Participants

We analyzed three physical education teachers (1 man and 2 women) with more than 2 years' experience who taught a total of 65 students with a mean age of 10.7 years. The students were from 3 years at different schools and included 26 first-second class students, 19 fifth-class students, and 20 sixth-class students).

This study was carried out in accordance with the recommendations of Ethical Committee of the University of La Laguna (Spain) with written informed consent from all subjects. All subjects gave written informed consent in accordance with the Declaration of Helsinki.

### Observation instrument

We used an *ad hoc* observation instrument (Anguera et al., 2007), called Analysis of Educational Discourse in Physical Education, or ADDEF as per its Spanish acronym (Table 1). The instrument was designed to discriminate between and record discursive strategies used by physical education teachers. It was suited to the multidimensional design of the study, and consisted of a combined field format and category system, which is the most recommendable system for studies of this type (Lacy and Darst, 1985; Castañer et al., 2013; Portell et al., 2015). We built a category system for each of the three dimensions or criteria derived from Coll and Onrubia's (2001) social constructivist theory regarding discursive strategies for the classroom: (1) exploration and activation of previous knowledge, (2) attribution of positive meaning by students to the concepts being taught, and (3) progressive establishment of increasingly expert and complex representations of the subject matter. Table 2 shows the three category systems, which are formed, respectively by three, seven, and four exhaustive, mutually exclusive categories.

**Table 1:** ADDEF Observation Instrument.

**Criterion 1. Exploration and activation of previous knowledge**
*Use of social framework (A1)*
References to social situations/events (or their meanings) related to the subject matter or task at hand with the aim of establishing sharing meanings in relation to these situations/events. Example: You have to jump like a frog
*Use of specific framework (A2)*
References to specific previously shared learning experiences, clearly highlighting their relationship with the subject matter or task at hand, seeking to establish shared meanings. Example: At the beginning of the course we practiced moving from one point to another; today we are going to do sprints.
*Request for information (A3)*
Use of strategies to obtain relevant information from the students on the subject matter or task at hand, but without mention of a social or specific framework. Example: How many different ways did they throw the ball?
**Criterion 2. Attribution of positive meaning by students to the concepts being taught**
*Use of meta-statements before the task (B1)*
References to what is going to be done or to what might occur, without linking these to a previous activity, and only including messages that refer to the subsequent learning activity. Example: We are going to play the 10-pass game so that the player who is about to receive the ball in movement learns to get free.
*Use of meta-statements during the task (B2)*
References that remind students about the goal of the task, i.e., about what it is they are trying to improve. Example: We are practicing our aim and learning to move the cones.
*Incorporation of students' contributions into the teacher's discourse (B3)*
Literal or near-literal incorporation into the teacher's discourse of elicited or spontaneous verbal contributions from the students about what they are learning. Example: As Laura says, I have to move faster.
*Incorporation of students' actions into the teacher's discourse (B4)*
Incorporation into the teacher's discourse of a specific aspect of a student's motor behavior, with specific reference to the student involved, with the aim of guiding learning. Example: Did you see how Luis moves his feet when skipping?
*Characterization of knowledge as something shared (B5)*
References to the subject matter or the task at hand, or their results, systematically using the first person plural (we), and drawing attention to what has been learned or is about to be learned, with the inclusion of a positive evaluation. Example: We have successfully kept the ball in the air.
*Acknowledgment of acquired personal knowledge (B6)*
References to current tasks or their results using the second or third person singular or plural (you, he/she, they) and highlighting something that has been learned. Example: Sandra, your shot was very good; you positioned your hands and feet just like we said you should earlier.
*Praise for verbal contribution or action (B7)*
References to current activities or their results using the second or third person singular or plural (you, he/she, they) in response to a motor behavior or verbal comment by a student or group of students, but without mention of a specific type of learning. Examples Very good! Nice! Perfect! Great! Excellent!
**Criterion 3. Progressive establishment of increasingly expert and complex representations of subject matter**
*Re-elaboration of student contributions (C1)*
Re-elaboration of a spontaneous or elicited motor or verbal contribution from a student, where the teacher expands, develops, reorganizes, trims, or corrects the relevant information. Example: Michael says that if we throw the ball in the air, we push our bodies upwards, and if we throw it in front of us, we push our bodies forwards.
*Characterization and labeling of aspects of content or context C2*
Redefinition and characterization of a concept, contextual aspects, an activity or its results; the teacher may do this spontaneously or use labels typically employed by the students. Example: The leg in front is called the drive leg.
*Introduction of different referential expressions (C3)*
Introduction of new referents (spatial, temporal, tactical-strategic, biomechanic-technical and/or physical-physiological) in relation to the task the students are about to start, or to an object or concept. The task/object/concept is clearly identified and highlighted. Example: When running in a hurdle race, it's not a good idea to jump over the hurdle when you are very close to it, as we can hurt ourselves. We are going to try to do it at a fast pace, with our front leg in a semi-bent position.
*Cognitive transfer of learning to a future situation (C4)*
Description and/or justification of how the object of the lesion or task can be applied in a future situation. Example: We are going to work on our spatial-temporal perception, and this will help us to know whether we can cross the road safely or not when we see a car coming.

**Table 2:** Number and percentage of discursive strategies used before and after participation in the collaborative action research program.

**Teachers**	**Teacher #1**				**Teacher #2**				**Teacher #3**			
**Phases**	**Pre-training**		**Post-training**		**Pre-training**		**Post-training**		**Pre-training**		**Post-training**	
**Categories (see Table 1)**	**No**.	**%**	**No**.	**%**	**No**.	**%**	**No**.	**%**	**No**.	**%**	**No**.	**%**
A1 (social framework)	20	6.49	34	6.59	4	1.41	6	1.34	2	1.57	0	0.00
A2 (specific framework)	11	3.57	24	4.65	10	3.52	10	2.23	3	2.36	1	0.33
A3 (request for information)	53	17.21	175	33.91	64	22.54	143	31.85	62	48.82	149	48.53
B1 (meta-statements before task)	4	1.30	4	0.78	1	0.35	11	2.45	0	0.00	1	0.33
B2 (meta-statements during task)	3	0.97	2	0.39	1	0.35	10	2.23	0	0.00	0	0.00
B3 (incorporation of students' contributions)	28	9.09	111	21.51	31	10.92	73	16.26	25	19.69	79	25.73
B4 (incorporation of students' actions)	2	0.65	11	2.13	0	0.00	10	2.23	0	0.00	0	0.00
B5 (characterization of knowledge as shared)	0	0.00	1	0.19	0	0.00	19	4.23	0	0.00	0	0.00
B6 (acknowledgement of acquired knowledge)	1	0.32	26	5.04	3	1.06	35	7.80	0	0.00	0	0.00
B7 (praise for verbal contribution/action)	169	54.87	82	15.89	161	56.69	69	15.37	16	12.60	36	11.73
C1 (re-elaboration of student contribution)	8	2.60	40	7.75	8	2.82	33	7.35	18	14.17	33	10.75
C2 (characterization/labeling of content/context)	4	1.30	0	0.00	0	0.00	11	2.45	1	0.79	7	2.28
C3 (introduction of referential expressions)	2	0.65	6	1.16	0	0.00	7	1.56	0	0.00	1	0.33
C4 (cognitive transfer to future situation)	3	0.97	0	0.00	1	0.35	12	2.67	0	0.00	0	0.00
Total	308	100	516	100	284	100	449	100	127	100	307	100

### Recording instrument

**Figure 1:**
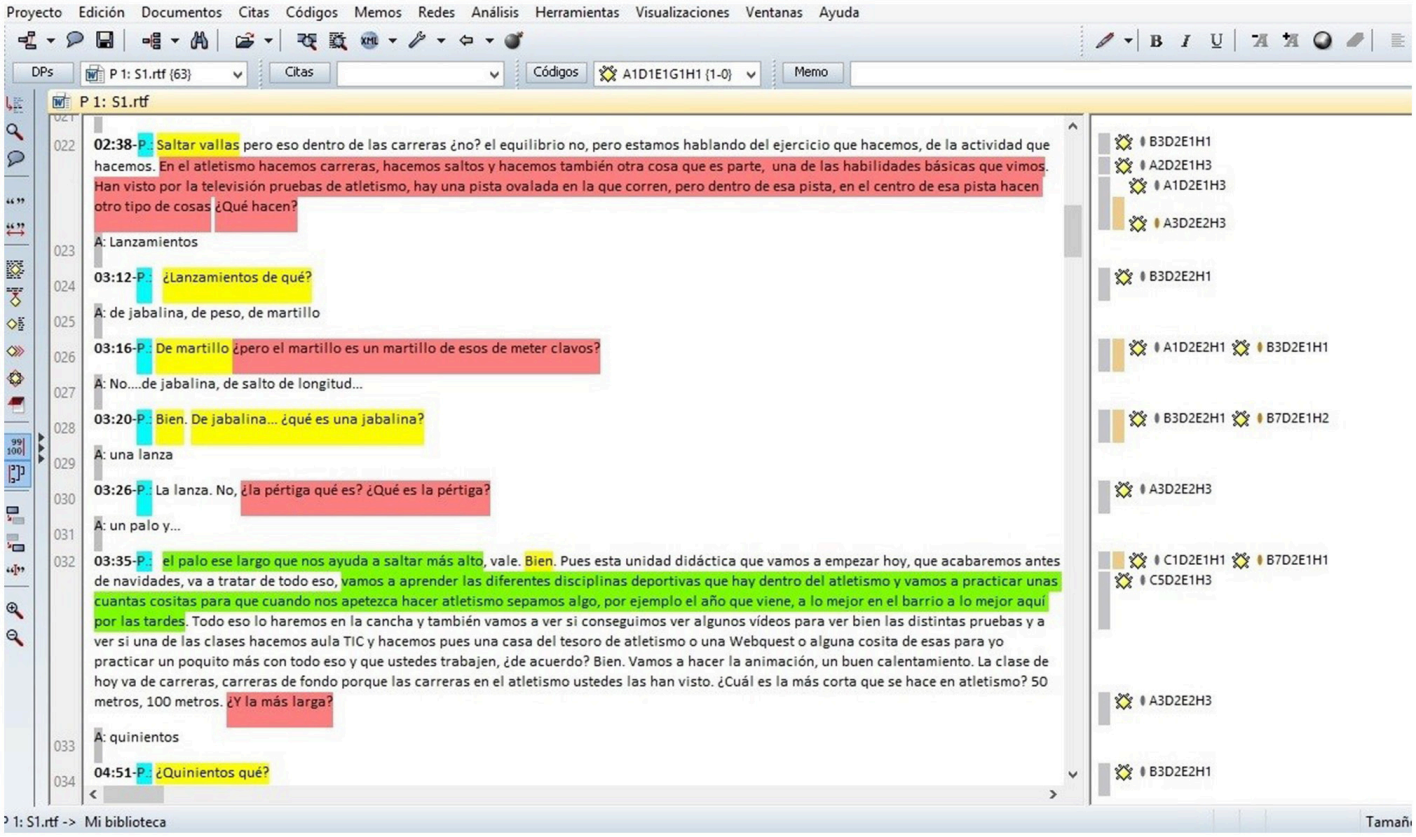
Screenshot of the data annotation process in ATLAS.ti.

The transcripts of the teachers' lessons were coded using the qualitative analysis program Atlas.ti v. 7.1.8. (Figure 1) Lag sequential analysis was performed in GSEQ 5.1 (Bakeman and Quera, 2011).

### Procedure

For the data collection stage, 29 sessions corresponding to two teaching modules were recorded. The first module (consisting of six sessions for teacher #1, four sessions for teacher #2, and five sessions for teacher #3) was taught before the collaborative action research program and the second module (consisting of five sessions for teacher #1, five sessions for teacher #2, and four sessions for teacher #3) was taught after the program. A total of 1,991 messages containing the discursive strategies analyzed were recorded: 719 before the program and 1,272 afterwards. All the sessions were recorded using a Panasonic HDC-HS100 video camera fitted with a wireless audio recording system (AKGPR81 + PT81).

The intraobserver reliability of the data was checked using Krippendorf's canonical agreement coefficient (Krippendorf, 2004), which is an adaptation of Cohen's kappa statistic (Cohen, 1960), used to analyze at least three datasets collected at three different points in time. The analysis was performed in HOISAN (v. 1.6.3.3) (Hernández-Mendo et al., 2012). Interobserver reliability was tested by having each of the three observers code a randomly selected segment of 15 min on three occasions, separated by 10 days each. The results yielded a mean kappa coefficient of 0.97, indicating almost perfect agreement. The reliability of the data was also guaranteed by applying the consensus agreement method (Arana et al., 2016), which is a qualitative method in which observers agree on how to code a particular item before it is included in the dataset. The three observers were trained for over 80 h over a 6-month period and recorded 15% of the total session content using the consensus agreement method.

### Data analysis

Because the first objective, which was quantitative in nature, consisted of identifying the verbal behavior of the participating teachers, the dataset of events recorded during each session was processed using lag sequential analysis. This data analysis technique, proposed by Bakeman (1978), and subsequently extended by Bakeman and Gottman (1986) and Bakeman and Quera (2011), has proven to be highly effective in diverse fields (Lapresa et al., 2013; Roustan et al., 2013), and is extremely useful for analyzing datasets compiled from direct and/or indirect observation that contain sequences of behaviors coded using an *ad hoc* observation instrument. The first step in this analysis is to define our *criterion behaviors* (the starting point of any possible patterns detected) and to apply the time lags defined for the study. Observed probabilities were calculated for each of the lags using the binomial test; this test produces adjusted residuals (Allison and Liker, 1982), which show the strength of association between significantly associated categories (i.e., between criterion behaviors and the conditional behaviors with which they are associated). The level of significance was set at *p* < 0.05. Adjusted residuals are prospective when the lags are analyzed in a forward direction from the criterion behavior (lags +1, +2, etc.) and retrospective when they are analyzed in a backward direction (lags −1, −2, etc.). Adjusted residual values higher than 1.96 and lower than 1.96 are therefore statistically significant. In this study, we looked at two retrospective lags (−2, −1) and two prospective lags (+1 and +2). In other words, we looked at the two events that occurred immediately before the criterion behavior and the two events that occurred immediately afterwards.

We also performed a descriptive statistical analysis of the number and percentage of discursive strategies used during the two teaching modules analyzed (Table 2).

## Results

### Descriptive analysis

Table 2 shows the descriptive statistics for the discursive strategies observed for each teacher before and after participation in the action research program.

An increase in the frequency and variety of discursive strategies employed by the teachers was observed in the post-training phase, indicating that participation in the collaborative action research program provided the teachers with a greater repertoire of discourse tools and resources with which to construct knowledge with their students.

### Detection of communication patterns

Tables 3–**5** show the adjusted residual values for the retrospective lags (−1, −2) and the prospective lags (+1, +2) for teachers #1, #2, and #3, respectively, before and after participation in the collaborative action research program (pre- and post-training). The first cell in each row shows the criterion behavior, while the remaining cells show the respective conditional behaviors and the corresponding adjusted residuals.

**Table 3:** Adjusted residuals for teacher #1 at the four lags analyzed before and after the collaborative action research program.

	**Lag**−**2**		**Lag**−**1**		**Lag 1**		**Lag 2**	
	**Pre-training**	**Post-training**	**Pre-training**	**Post-training**	**Pre-training**	**Post-training**	**Pre-training**	**Post-training**
A1	A2 2.88 C3 2.52		A2 2.81 B6 3.76		B6 3.87		A3 2.03 C2 3.7	B5 3.73
A2		B1 2.03 B6 2.83		B1 4.41 B6 2.75	A1 2.81 C3 3.51		A1 2.88	
A3	A1 2.03		B1 3.86	B3 2.27	B3 4.82 C1 2.49	B3 5.86 C1 6.08	B6 2.17 C1 2.44	
B1			B1 3.44		A3 3.86	A2 4.41	B3 3.56	A2 2.03B4 3.14
B2		B6 2.88					C4 5.62	B7 3.25
B3	B1 3.56		A3 4.82	A3 5.86	B1 3.44 C1 2.79	A3 2.27		
B4		B1 3.14				B7 4.35		
B5		A1 3.73		B7 2.31				
B6	A3 2.17	B7 3.75	A1 3.87	B7 3.79	A1 3.76	A2 2.75 B7 2.65		A2 2.83B2 2.88
B7		B2 3.25		B4 4.35 B6 2.65		B5 2.31B6 3.79		B6 3.75
C1	A3 2.44	B3 2.6	A3 2.49 B3 2.79	A3 6.08			C4 3.29	
C2	A1 3.7							
C3			A2 3.51			B7 3.4	A1 2.52	
C4	B2 5.62 C1 3.29							

*Adjusted residual values >1.96 implies p < 0.05*.

For teacher #1 in the pre-training phase, a strong, stable association was observed between category A1 (social framework) and acknowledgment of acquired knowledge (B6, adjusted residual = 3, 87) and request for information (A3) at lag 2 (adjusted residual = 2.03) (Table 3).

Example:

*Teacher:* You have two weights and two discs over there, but be careful as it is very heavy. It's made of very hard rubber like the rubber on trucks (A1). You picked that up really well Jorge with your hands, opening your fingers (B6). How do you all think we can throw this weight? (A3).

This indicates that teacher #1 tends to ask questions immediately after making a comment linking the subject matter or task to everyday, social aspects. Requests for information (A3) were predominantly followed by incorporation of student contributions into the teacher's discourse (B3, adjusted residual = 4.82) or re-elaboration of contributions (C1, adjusted residual = 2.49).

Example:

*Teacher:* What do we need to take into account in a race that lasts for a long time? (A3).
Student: Speed.
*Teacher:* Speed (B3). What do we do with speed Alba? (A3).
Student: Control it.
*Teacher:* Control it, spread out our energy (C1).

The above exchange shows a pattern formed by a question that triggers an answer, which is repeated and then elaborated on.

The pattern observed for teacher #2 (Table 3) was very similar, with requests for information strongly associated with incorporation of contributions (B3, adjusted residual = 8.81) and re-elaborations (C1, adjusted residual = 4.42).

Example:

*Teacher:* Sandra, tell me one way of warming up (A3).
Student: Heels back.
*Teacher:* Heels back (B3).

In this case, reference to the social framework (A1) was slightly more strongly associated with the use of meta-statements during task execution (B2, adjusted residual = 8.32), indicating that the teacher's strategy was to link the learning objective to sociocultural aspects. The social framework was also associated, but to a lesser extent, with re-elaborations (C1, adjusted residual = 2.68).

Example:

*Teacher:* If the person holding it touches you, you have to stand on one leg, as if you were a stork (A1).
Student: Like in the red cross game.
*Teacher:* Yes, like in the red cross game but here you can be freed and saved (C1).

The teacher shows concern for establishing links between what the students already know and what is being taught. She links concepts from the animal world to the rules of the game to help the students to understand them. In the case of teacher #3, requests for information were also associated with re-elaborations (C1, adjusted residual = 3.06), showing a desire to explore and build on previous knowledge. Labeling (C2) was also associated with a literal incorporation of the students' contributions into the discourse of teacher #3 (B3, adjusted residual = 2.03).

For teacher #1, the association observed in the pre-training phase between requests for information (A3) preceded by B3 (adjusted residual = 5.86) and C1 (adjusted residual = 6.08) was even stronger in the post-training phase, showing that the teacher continued to use this discursive pattern as a means of constructing knowledge (Table 3).

Example:

*Teacher:* They are practicing techniques. Which ones? (A3).
Student: Dodging.
*Teacher:* Dodging, dribbling, and feinting (C1).

The teacher constantly interacts with the students by asking them questions, acknowledging their answers, and then elaborating on them for the benefit of the group. We also observed a new association between the use of meta-statements (B1) and a specific framework (A2) at lag 1 (adjusted residual = 4.41) and lag 2 (adjusted residual = 2.03).

Example:

*Teacher:* Now we are going to learn how to pass the ball with the stick and to shoot. (B1). Does anyone remember how to hit the ball; we saw it yesterday? (A2).

The above example shows the use of a new discursive strategy involving commenting on the learning objective and linking it to a previous shared experience, thereby aiding comprehension. The teacher also incorporated the students' actions into his communication (B4) and combined this with praise (B7, adjusted residual = 4.35).

Example:

*Teacher:* Look how Carlos is holding the stick (B4). Good Miguel (B7), Good Luis (B7).

We also observed a recurrent pattern consisting of the prospective and retrospective interlinking of praise (B7) and recognition (B6), indicating concern for creating a positive learning climate.

Example:

*Teacher:* Nice Carlos (B7), good pass Dailos (B6).

The teacher also praised the students when comments were made by the group (B5, adjusted residual = 2.31). The above observations strongly suggest that participation in the collaborative action research program led teacher #1 to adopt new discursive strategies as a means of constructing knowledge.

The number of significant associations between the discursive strategies analyzed was also higher for teacher #2 in the post-training phase (Table 4). First, the social framework (A1) was strongly associated with labeling (C2, adjusted residual = 2.25).

**Table 4:** Adjusted residuals for teacher #2 at the four lags analyzed before and after the collaborative action research program.

	**Lag**−**2**		**Lag**−**1**		**Lag 1**		**Lag 2**	
	**Pre-training**	**Post-training**	**Pre-training**	**Post-training**	**Pre-training**	**Post-training**	**Pre-training**	**Post-training**
A1		B6 2.66		A2 2.4 C2 2.4	B2 8.32 C1 2.68	B1 2.25 B2 2.4 C2 2.25		B1 2.23
A2	C1 3.28C4 5.17	B7 3.4	C4 5.21	B7 3.39		A1 2.4	C4 5.17	B1 3.58
A3			B3 2.68	C1 2.5	B3 8.81 C1 4.42	B3 5.61 C1 4.59		B5 2.15
B1		A1 2.23 A2 3.58		A1 2.25 B5 2.51			B3 2.87	B6 2.15
B2			A1 8.32	A1 2.4 C2 3.61		B7 2.19		B7 3.05
B3	B1 2.87	B5 2.11	A3 8.81	A3 5.61	A3 2.68			
B4								
B5		A3 2.15				B1 2.51		B3 2.11
B6		B1 2.45 B7 2.89 C3 3.5		B7 2.87				A1 2.66
B7		B2 3.05		B2 2.19		A2 3.39 B6 2.87		A2 3.4 B6 2.89
C1		C4 2.5	A1 2.68 A3 4.42	A3 4.59		A3 2.5	A2 3.28	
C2				A1 2.25		A1 2.4 B1 3.61		
C3						B2 2.16	A2 5.17	B6 3.5
C4	A2 5.17				A2 5.21			C1 2.58

*Adjusted residual values >1.96 implies p < 0.05*.

Example:

*Teacher:* It's shaped like Indian feathers (A1) but it's not a duster, it's called a shuttlecock or an *indiaca* (C2).

A1 was also associated with the use of meta-statements before (B1, adjusted residual = 2.25) and during the task (B2, adjusted residual = 2.4), as well as with A2 at lag −1 (adjusted residual = 2.4), showing that the teacher actively linked aspects of the task at hand to sociocultural content. The previously observed pattern between requests for information (A3) and incorporation of students' contributions (B3) and re-elaborations (C1) was still present but stronger (adjusted residual = 5.61 and adjusted residual = 4.59, respectively). Finally, incorporation of new referential expressions (C3) was associated with the use of meta-statements during the task (B2, adjusted residual = 2.16) indicating a concern for highlighting the important aspects of the task at hand.

Example:

*Teacher:* If you are going to shoot hard, stand away from the wall a little, look at the distance and think about how hard you are going to kick the ball (C3) and remember that we are practicing shooting and receiving in this task (B2).

The stronger associations observed between categories and the greater number of patterns suggest that this teacher intentionally incorporated a greater range of strategies into his teaching.

In the post-training stage, teacher #3 (Table 5) continued to use the communication pattern consisting of requests for information followed by incorporation of student contributions (B3, adjusted residual = 6.28).

**Table 5:** Adjusted residuals for teacher #3 at the four lags analyzed before and after the collaborative action research program.

	**Lag**−**2**		**Lag**−**1**		**Lag 1**		**Lag 2**	
	**Pre-training**	**Post- training**	**Pre-training**	**Post-training**	**Pre-training**	**Post-training**	**Pre-training**	**Post-training**
A1							C1 2.36	
A2								
A3				B3 3.32 C2 1.9	C1 3.06	B3 6.28		
B1								
B2								
B3				A3 6.28	C2 2.03	A3 3.32		A3 2.37
B4								
B5								
B6								
B7				C3 2.73			C2 2.62	
C1	A1 2.36 C2 2.36		A3 3.06					
C2	B7 2.62	B3 2.03				A3 1.99	C1 2.36	
C3						B7 2.73		
C4								

*Adjusted residual values >1.96 implies p < 0.05*.

Example:

*Teacher:* What do you know about baseball?
*Student:* You have to bat the ball.
*Teacher:* You have to bat the ball (B3). And what else? (A3).
*Student:* Be fast.
*Teacher:* Be fast (B3).

In the pre-training phase, there was a significant association between A3 and C1, while in the post-training phase; there was a significant association between A3 and B3. Fewer associations were observed between discursive strategies for this teacher than for teachers #1 and #2.

## Discussion

We have studied the verbal behavior of three teachers in their natural setting. Although each of these teachers is considered as a “single case,” they were monitored intensively over a series of sessions, resulting in the generation of large volumes of data, which, once converted into matrices of codes through annotation in ATLAS.it, were analyzed by lag sequential analysis to uncover patterns related to the use of social constructivist communication strategies. We are particularly interested in determining the extent to which single cases can reveal patterns that can then be merged, either partially or fully, to methodologically advance toward a multiple case, as proposed by Stake (2006) and Yin (2014).

We wished to investigate whether participation in a collaborative action research program would result in significant changes in the use of discursive strategies of a social constructivist nature by physical education teachers. Our analysis of these strategies by primary school physical education teachers shows a clear pattern composed of questions-answers-literal incorporation-re-elaboration of students' answers both before and after participation in a collaborative action research program designed to improve familiarity with and use of constructivist discursive strategies as a methodological resource. Such strategies encourage students to engage more actively in their learning, as claimed by Cazden (1988), Lemke (1990), Lobato et al. (2005) and Wright and Forrest (2007), who highlighted the importance of the triadic IRE dialogue pattern. The recurrent discursive pattern observed in our study (request for information (A3) + incorporation of students' contributions (B3), like request for information (A3) + re-elaboration of student contribution (C1), which is similar to the ERE pattern (Bowers and Nickerson, 2001), provides teachers with the means to guide their students toward the construction of significant meaning through the use of questions, reasoning, and argumentation. In this case, evaluation of students' answers leads teachers to take two decisions, i.e., to incorporate what the students say into their discourse and to re-elaborate when the answer is incomplete. Use of questioning to promote learning has been advocated by many authors (Wallian and Chang, 2007; Harvey and Light, 2015), who have shown that the use of open-ended questions in the classroom encourages reflective learning (Dyson et al., 2004; Mitchell et al., 2006; Hubball et al., 2007). Similarly, teachers who use closed questions to control construed meanings are better positioned to guide and elaborate on answers and to draw students' attention to the relevance or importance of certain learning points. Such strategies have been shown to play an important role in aiding understanding (Webster et al., 2012, 2013). In our analysis, just one change in the use of discursive strategies was observed for teacher #3 following his participation in the collaborative action research program. The observation of additional associations: meta-statements before task (B1) + request for information (A3) and incorporation of students' contributions (B3) + meta-statements before task (B1) in the pre-training phase for teacher #1 shows that this teacher was already using some of these strategies, even though he was not familiar with the theory behind them.

Participation in the collaborative action research program appears to have had a positive impact on teaching performance, as we detected an increase in the number and strength of associations observed in the post-training phase, suggesting that the use of new communication patterns was both intentional and strategic. The fact that the teachers recognized the usefulness of the strategies is evident through statements such as: “I can see that the kids are improving. I think that they are understanding things better and are doing the exercises with a greater understanding of why they are doing them and they are also making an effort to do things a little better, this gives me the strength to keep doing things and to keep trying. It's mutual reinforcement.” They acknowledged the advantages of using constructivist techniques, probably because they feel that they will make their work easier and help their students to learn better. The patterns detected show that the teachers prefer to explore students' knowledge and reinforce correct answers rather than advance this knowledge to a more expert form; one exception is the use of re-elaborations of student contributions in the post-training phase. This greater tendency to explore and reinforce learning may be related to the short duration of the teaching modules analyzed. The identification of stable sequences in the forms of patterns as opposed to the use of isolated categories in the pre-training phase may indicate that the associations observed between categories from criteria 1 and 2 in the observation instrument reflects acquired practices, or habits, rather than an intentional, strategic use of strategies grounded in theoretical knowledge. The post-training results, by contrast, show that the teachers were familiar with the theory underlying the strategies they were incorporating into their instructional communication. It would therefore appear that participation in the collaborative action research program equipped the teachers with a greater repertoire of discursive strategies to actively engage students in the joint enterprise of learning.

We found that the three teachers all modified their use of discursive strategies after participation in the program. Particularly noticeable were improvements in the use of praise (B7), which was associated with incorporation of students' actions (B4) and acknowledgement of acquired knowledge (B6) as forms of recognition during task execution in the case of teacher #1. This observation reflects an increased interest in creating a positive learning climate. The continuous linking of previous knowledge is necessary to build knowledge, and in the post-training phase, teacher #2 intentionally used patterns linking social frameworks (A1) to other categories, such as meta-statements before task (B1) and meta-statements during task (B2). We also observed a significant relationship between characterization and labeling (C2) with explanations of tasks (B1).

Participation in the collaborative action research program also brought about changes in the way the teachers communicated with their students, as reflected in comments such as “I can see better results, I have saved time, and I feel that I am communicating better with my students. I can use discursive strategies to improve my teaching.” The incorporation of new strategies also indicates the teachers' concern for improving both the teaching and learning process. Our findings support the usefulness of collaborative action research programs as an effective means of perfecting teaching performance.

Discursive strategies, which involve the conscientious use of language, should be used both strategically and intentionally in the classroom. Teachers need to know which form of language to use and when, and to see discursive strategies as a methodological resource rather than a means of support for their teaching activities. Teachers who use discursive strategies are thus effectively incorporating the potential of a scientific theory into their teaching practice and linking this to academic content. The integration of different formal and informal learning processes is particularly important in competence-based learning that aims to help students relate learning strategies to content and to use them effectively in different situations and contexts.

The limitations of our study are largely related to the difficulties associated with working with verbal behavior, as there is a risk of drawing inferences from the theoretical framework used as a reference for building the observation instrument.

The results of this study should bring us to reflect on the effectiveness of the methodological resources we conscientiously use in the classroom and on the functionality of the discursive strategies used by physical education teachers.

## Conclusions

The teachers showed a consistent use of constructivist discursive strategies before and after participation in a research action program. The pattern detected consisted of requests for information followed by the incorporation of the students' contributions into their communication and the re-elaboration of their answers.

Following participation in this program, the teachers were seen to use more discursive strategies, generating new patterns.

By using lag sequential analysis, we were able to uncover hidden yet solid, meaningful patterns in the instructional communication of physical education teachers and to generate information of potential value for both teachers and researchers.

## Author contributions

AG-F developed the project and supervised the design of the study and the drafting of the manuscript. He was responsible for data collection and handling, critically revised the content, performed the lag sequential analysis, and wrote the method section. FJ-J was responsible for reviewing the literature and drafting the manuscript. MTA collected and analyzed the data and supervised the drafting of the manuscript. All authors approved the final, submitted version of the manuscript.

### Conflict of interest statement

The authors declare that the research was conducted in the absence of any commercial or financial relationships that could be construed as a potential conflict of interest. The reviewer, MB, declared a shared affiliation, though no other collaboration, with one of the authors, MTA, to the handling Editor.
